# Supplementary material for: Association between sense of coherence and depression in patients with chronic pain: A systematic review and meta-analysis
Source: PLoS One. 2023 Jan 11;18(1):e0279959. doi: 10.1371/journal.pone.0279959 (PMC9833581; doi:10.1371/journal.pone.0279959)
Supplement: Attachment — Submitted filename: Response to Reviewers -3108.docx [file pone.0279959.s002.docx]

## PONE-D-21-14267 Association between sense of coherence and depression in patients with chronic pain: a systematic review and meta-analysis. PLOS ONE

1. Please ensure that your manuscript meets PLOS ONE's style
requirements, including those for file naming. The PLOS ONE style
templates can be found at
<https://journals.plos.org/plosone/s/file?id=wjVg/PLOSOne_formatting_sample_main_body.pdf>
and
<https://journals.plos.org/plosone/s/file?id=ba62/PLOSOne_formatting_sample_title_authors_affiliations.pdf>

Thank you for the information. It’s checked and done.

2. Thank you for stating the following in the Acknowledgments Section of
your manuscript:

"We wish to thank the Primary Care Prevention and Health Promotion
Network (RedIAPP Health Institute Carlos III, Spain); Research Group
B21_R17 of the Department of Science, University and the Knowledge
Society of the Government of Aragon (Spain) and Feder Funds "Another way
to make Europe".

We note that you have provided funding information that is not currently
declared in your Funding Statement. However, funding information should
not appear in the Acknowledgments section or other areas of your
manuscript. We will only publish funding information present in the
Funding Statement section of the online submission form.
Please remove any funding-related text from the manuscript and let us
know how you would like to update your Funding Statement. Currently,
your Funding Statement reads as follows:

"This work was supported by Carlos III Health Institute grant number
PI18/01336, Feder Funds "Another way to make Europe". The funders had no
role in study design, data collection and analysis, decision to publish,
or preparation of the manuscript."

Please include your amended statements within your cover letter; we will
change the online submission form on your behalf

Thank you. We have eliminated the funding information of the “Acknowledgments” section. Now it is stated as follows:

We wish to thank the Primary Care Prevention and Health Promotion Network (RedIAPP Health Institute Carlos III, Spain) and Research Group B21_R17 of the Department of Science, University and the Knowledge Society of the Government of Aragon (Spain).

3. PLOS requires an ORCID iD for the corresponding author in Editorial
Manager on papers submitted after December 6th, 2016. Please ensure that
you have an ORCID iD and that it is validated in Editorial Manager. To
do this, go to 'Update my Information' (in the upper left-hand corner of
the main menu), and click on the Fetch/Validate link next to the ORCID
field. This will take you to the ORCID site and allow you to create a
new iD or authenticate a pre-existing iD in Editorial Manager. Please
see the following video for instructions on linking an ORCID iD to your
Editorial Manager account: <https://www.youtube.com/watch?v=_xcclfuvtxQ>

Thank you. We cannot do it. The warning message stated: “there is already an identical orcid registered”. It is because she has another account with another email. Her ORCID is 0000-0001-6565-9699. We wrote you asking if it is possible to unify the two accounts. We are still waiting for the response. Thank you in advance.

4. We note that this manuscript is a systematic review or meta-analysis;
our author guidelines therefore require that you use PRISMA guidance to
help improve reporting quality of this type of study. Please upload
copies of the completed PRISMA checklist as Supporting Information with
a file name "PRISMA checklist"

Our research is a Meta analysis of observational studies, so we have completed the MOOSE (Meta-analyses Of Observational Studies in Epidemiology) Checklist, as it is stablished and cited in the paper (*Supporting information - S1 File. MOOSE (Meta-analyses Of Observational Studies in Epidemiology) Checklist*.).

5. Please include captions for your Supporting Information files at the
end of your manuscript, and update any in-text citations to match
accordingly. Please see our Supporting Information guidelines for more
information: <http://journals.plos.org/plosone/s/supporting-information>.

Thank you. Now it is done.

6. We noticed you have some minor occurrence of overlapping text with
the following previous publication(s), which needs to be addressed:

- <https://www.mdpi.com/2071-1050/11/4/1115/html>

The text that needs to be addressed involves the Limitations section.

In your revision ensure you cite all your sources (including your own
works), and quote or rephrase any duplicated text outside the methods
section. Further consideration is dependent on these concerns being
addressed.

Thank you. We have rewritten the entire paragraph.

**Response to Reviewers**

**Reviewer #1**: Thank you for the opportunity to review this work. It is interesting to see the potential relationship between sense of coherence and depression in those with chronic pain conditions. The concept of sense of coherence in relation to pain and depression is really interesting and significant to help refine interventions to assist these comorbid conditions of chronic pain and depression. The authors described the process and significance of this work very clearly and thoroughly. One curiosity I had as I read this work was the overlap between the concepts of sense of coherence, resilience, and spiritual wellbeing research--particularly the body of research on meaning and purpose. I appreciated the authors inclusion of spirituality as a mechanism and contributor to sense of coherence, as well as resilience. However, I do wonder if a potential limitation in this review and meta-analysis has to do with these overlapping concepts of sense of coherence, resilience, and meaning and purpose. Due to their overlapping nature, the research for each concept seems to be siloed so that there might be the possibility of researchers/clinicians speaking about parallel concepts and not understanding the relationships and significance to each other and to therapeutic interventions.

**Answer reviewer 1:** Thank you very much for your thorough review. We are very grateful for your comments and feedback. We would like to note that with the study being a systematic review and meta-analysis about two very specific concepts and questionnaires (Sense of Coherence, SOC – measured specifically by SOC-13 and SOC-29 – and depression), we needed to put aside the related concepts that you mentioned and narrow the search. Although we looked into it, finally, we chose to work with SOC as it is the core concept in the salutogenesis theory developed by Antonovsky (1996). This was due to its relevance, as well as the fact that the SOC questionnaire has been widely used and translated into at least 33 languages, and also seems to be a reliable, valid, and cross-culturally applicable instrument that measures how people manage stressful situations and stay well (with these three domains: comprehensibility, manageability, and meaningfulness). We also wrote about possible therapies that increase SOC, however, of course, these methods can also serve to increase resilience or other concepts with similar functions. We agree with you and recognize that in practice these concepts overlap. As you have said, relying only on one concept at a time, separately, can be a limitation.

As such, we have found more literature in this regard and added your compelling suggestion in the “Introduction” and “Limitations” sections:

INTRODUCTION

“The positive association between religious/spiritual beliefs and sense of coherence deserves further examination to promote a multidimensional approach in its study, as shown in another Cretan study (Stefanaki et al., 2014). This deserves attention as a potential explanatory mechanism regarding the association between depression and SOC (Anyfantakis et al., 2015), as it has been reported that religious and spiritual involvement may help patients to cope better with stressful situations by providing meaning and hope, and also may result in healthier lifestyle practices (Bonelli et al., 2012).”

LIMITATIONS

“A general limitation is the intrinsic flaws that meta-analytic studies contain (Rosenthal & DiMatteo, 2001). We note that most of the studies identified in our review had cross-sectional or longitudinal designs (Bäck et al., 2019; Duvdevany et al., 2011; Hållstam et al., 2016, 2017; Pakarinen et al., 2015; Piekutin et al., 2018; Schnyder et al., 1999, 2000; Schrier et al., 2012) — excluding a Randomized Control Trial (RCT) (Nilsson & Ekberg, 2010). Therefore, we had to work with correlation coefficients, and, as such, directional or causality relationships could not be determined. Additionally, the heterogeneous results imply that additional research is needed to see if other moderating variables, such as the sample's level of education (Duvdevany et al., 2011; Lillefjell et al., 2015) and general health indicators of the sample (Konttinen et al., 2008), are important to the SOC-depression association. Besides this, SOC could be overlapped with other concepts with similar core dimensions, such as resilience, sense of coherence, hardiness, purpose in life, self-transcendence (Lundman et al., 2010), or meaning in life (Bartrés-Faz et al., 2018). Focusing only on one concept can result in missing other parallel concepts that have a certain significance and have an influence on therapeutic interventions.

Furthermore, a prior aim of this research was to examine the interaction between SOC and depression in the general population. This was not possible due to the variety of the samples and the lack of studies about the general population. As such, RCTs and longitudinal studies with general population samples that include the questionnaires in their measurements are needed. Furthermore, future research is needed to not only find out if there is a dominant influence of one variable on another but also clarify the relationship between SOC and chronic pain, and SOC and depression.”

**Reviewer #2**: The authors referenced a PROSPERO ID that described a systematic review of sense of "coherence and depression" from 2010 to 2020. This does not agree with the described protocol described in the manuscript that examines SOC and depression AND chronic pain. The authors should provide explicit descriptions of their inclusion/exclusion criteria for their review so that their findings can be replicated and confirmed.

Is SOC really an important factor in depression levels in people with chronic pain? The authors described/assessed the relationship between SOC and depression within the context of chronic pain. However, they provide no definitive data on this relationship. The authors describe the bidirectional nature of depression and chronic pain but make no mention of which variable has a dominant influence (if it exists) over the other. Perhaps chronic pain leads to lower sense of coherence and the relationship between depression and SOC is a by product of this. Perhaps then the authors can clarify further their interest/focus on the relationship between SOC and depression and not SOC and chronic pain with depression as a consequence of chronic pain or a consequence of lower sense of coherence.

**Answer reviewer 2:** Thank you very much for your thorough review. We are very grateful for your comments and feedback. First of all, we agree with you and have consequently started an update in PROSPERO, as we did not update the information there due to the fact that we were extremely focused on the paper. When the PROSPERO managers approve the new version, it will be exactly the same as in the paper. Thank you for advising us.

Regarding your question, the analysis of SOC and depression in patients with chronic pain is due to our interest in analyzing the relationship between SOC and depression, as this paper is part of a bigger RCT research project in which we assess both variables. However, given the heterogeneity of the articles found, and the variability in the etiology of depression, it was decided to analyze this relationship in chronic pain patients, as its study is more frequent. Moreover, the relationship between SOC and depression has been highly reported comparing the relationship of SOC and chronic pain (for example, several studies have confirmed that SOC is inversely related to depression, presenting a protective capacity against depressive symptoms (López-Martínez et al., 2019; Plata-Muñoz et al., 2004; Skärsäter et al., 2009).

As such, we have found more literature in this regard and added more information in the “Introduction” section, which we have taken from this paragraph:

“Individuals with stronger SOC take less medication, and assess pain as being less severe and having a lower effect on their mood. These individuals declared that they could control pain and have less often catastrophizing thoughts. Therefore, we can conclude that the role of SOC as a buffer in the functioning of sick individuals has been confirmed (Andruszkiewicz et al., 2017).”

However, as you have said, relying only on one specific relationship and in a definite direction can be a limitation. As such, we have rewritten the “Limitations” section:

“A general limitation is the intrinsic flaws that meta-analytic studies contain (Rosenthal & DiMatteo, 2001). We note that most of the studies identified in our review had cross-sectional or longitudinal designs (Bäck et al., 2019; Duvdevany et al., 2011; Hållstam et al., 2016, 2017; Pakarinen et al., 2015; Piekutin et al., 2018; Schnyder et al., 1999, 2000; Schrier et al., 2012) — excluding a Randomized Control Trial (RCT) (Nilsson & Ekberg, 2010). Therefore, we had to work with correlation coefficients, and, as such, directional or causality relationships could not be determined. Additionally, the heterogeneous results imply that additional research is needed to see if other moderating variables, such as the sample's level of education (Duvdevany et al., 2011; Lillefjell et al., 2015) and general health indicators of the sample (Konttinen et al., 2008), are important to the SOC-depression association. Besides this, SOC could be overlapped with other concepts with similar core dimensions, such as resilience, sense of coherence, hardiness, purpose in life, self-transcendence (Lundman et al., 2010), or meaning in life (Bartrés-Faz et al., 2018). Focusing only on one concept can result in missing other parallel concepts that have a certain significance and have an influence on therapeutic interventions.

Furthermore, a prior aim of this research was to examine the interaction between SOC and depression in the general population. This was not possible due to the variety of the samples and the lack of studies about the general population. As such, RCTs and longitudinal studies with general population samples that include the questionnaires in their measurements are needed. Furthermore, future research is needed to not only find out if there is a dominant influence of one variable on another but also clarify the relationship between SOC and chronic pain, and SOC and depression.”

While revising your submission, please upload your figure files to the
Preflight Analysis and Conversion Engine (PACE) digital diagnostic tool,
<https://pacev2.apexcovantage.com/>. PACE helps ensure that figures meet
PLOS requirements. To use PACE, you must first register as a user.
Registration is free. Then, login and navigate to the UPLOAD tab, where
you will find detailed instructions on how to use the tool. If you
encounter any issues or have any questions when using PACE, please email
PLOS at [figures@plos.org](mailto:figures@plos.org). Please note that Supporting Information files
do not need this step.
