# Supplementary material for: Association between sense of coherence and depression in patients with chronic pain: A systematic review and meta-analysis
Source: PLoS One. 2023 Jan 11;18(1):e0279959. doi: 10.1371/journal.pone.0279959 (PMC9833581; doi:10.1371/journal.pone.0279959)
Supplement: Attachment — Submitted filename: Response to Reviewers 02.docx [file pone.0279959.s003.docx]

## PONE-D-21-14267R1

## Association between sense of coherence and depression in patients with chronic pain: a systematic review and meta-analysis.

## PLOS ONE

**Comments to the Author**

1. If the authors have adequately addressed your comments raised in a previous round of review and you feel that this manuscript is now acceptable for publication, you may indicate that here to bypass the “Comments to the Author” section, enter your conflict of interest statement in the “Confidential to Editor” section, and submit your "Accept" recommendation.

Reviewer #1: All comments have been addressed

Reviewer #2: All comments have been addressed

Reviewer #3: (No Response)

2. Is the manuscript technically sound, and do the data support the conclusions?

The manuscript must describe a technically sound piece of scientific research with data that supports the conclusions. Experiments must have been conducted rigorously, with appropriate controls, replication, and sample sizes. The conclusions must be drawn appropriately based on the data presented.

Reviewer #1: Yes

Reviewer #2: Yes

Reviewer #3: No

3. Has the statistical analysis been performed appropriately and rigorously?

Reviewer #1: Yes

Reviewer #2: Yes

Reviewer #3: Yes

4. Have the authors made all data underlying the findings in their manuscript fully available?

The PLOS Data policy requires authors to make all data underlying the findings described in their manuscript fully available without restriction, with rare exception (please refer to the Data Availability Statement in the manuscript PDF file). The data should be provided as part of the manuscript or its supporting information, or deposited to a public repository. For example, in addition to summary statistics, the data points behind means, medians and variance measures should be available. If there are restrictions on publicly sharing data—e.g. participant privacy or use of data from a third party—those must be specified.

Reviewer #1: Yes

Reviewer #2: Yes

Reviewer #3: No

5. Is the manuscript presented in an intelligible fashion and written in standard English?

PLOS ONE does not copyedit accepted manuscripts, so the language in submitted articles must be clear, correct, and unambiguous. Any typographical or grammatical errors should be corrected at revision, so please note any specific errors here.

Reviewer #1: Yes

Reviewer #2: Yes

Reviewer #3: Yes

6. Review Comments to the Author

Please use the space provided to explain your answers to the questions above. You may also include additional comments for the author, including concerns about dual publication, research ethics, or publication ethics. (Please upload your review as an attachment if it exceeds 20,000 characters)

Reviewer #1: Thank you for your response to my questions and the additions to the manuscript. I think this helps provide greater context and significance to this work.

**Answer reviewer 1:** Thank you very much for your thorough review. We are very grateful for your comments and feedback.

Reviewer #2: The authors are to be commended on this important undertaking. The PROSPERO information needs to be updated prior to publication.

**Answer reviewer 2:** Thank you very much for your thorough review. We are very grateful for your comments and feedback. PROSPERO information has been updated.

Reviewer #3: Thank you for the opportunity to review this work. The work focused on the association between sense of coherence and depression in patients with chronic pain. It is interesting and valuable. However, the manuscript still has the following problems worthy of attention, through the improvement of these problems can better improve the quality of the manuscript.

**Answer reviewer 3:** Thank you very much for your thorough review. We are very grateful for your comments and feedback. Following your comments and suggestions, we have made changes to the manuscript, which we detail below, point by point.

Abstract:

1) The author should describe the publication time of the included literature and the search time of this study respectively. The current statement is easy to cause ambiguity.

**Answer reviewer 3:** Thank you. There were no restrictions regarding the publication date of the study since we knew that we would find few articles in general, since the topic was new. So, we have put this data in the abstract and in methodology since, as you say, it was not written. We have added the following sentence:

“There were no restrictions regarding the date of publication of the study.”

2) The authors do not search the Cochrane Library (CENTRAL) database, but this database is also important for meta-analysis.

**Answer reviewer 3:** Thank you. Our research is a Meta analysis of observational studies. And, as we put in the protocol: *The searches were carried out in PubMed, Web of Science, Embase, PsycINFO, Psicodoc, ScienceDirect and Dialnet*. We did not use the Cochrane Library (CENTRAL) database because we did not want to include other meta-analyses in our search; since, as we also put in the protocol: *Studies with underage individuals, non-indexed articles, systematic reviews and meta-analyses, theoretical studies, conference articles, insufficient data, single case studies and articles not written in English or Spanish were excluded*. In any case, following your suggestion, we have done a quick search to see what Cochrane results came out and we have not found anything relevant that we had not already taken into account. If you see it appropriate, we can add this search in methods. We are open to suggestions regarding this topic.

Introduction

1) It is necessary to make appropriate research hypothesis according to the current research situation.

**Answer reviewer 3:** Thank you. It is true that we have forgotten to add the hypothesis. We have already added it like this:

“The hypothesis of this study is that SOC and depression are associated in patients with chronic pain, regardless of their age and gender.”

Methods

1) The authors lack the necessary description of the method content. Although they have provided the registration number (PROSPERO- ID = CRD42020157512), it seems that the current system state does not provide key information about the method. Even if the authors use the PROSPERO system in their research, the manuscript should also provide the readers with necessary information for reference.

**Answer reviewer 3:** Thank you. We have reviewed what was written in PROSPERO and we have made sure that all the information written in PROSPERO is reflected in the manuscript. In addition, we have added the missing information as we have pointed out in the previous comments. If you think there is any more information missing about the methods, you can point it out, we are open to new suggestions.

2) Similarly, the manuscript lacks the description of the publication time of the included literature, the criteria for literature screening and other necessary information.

**Answer reviewer 3:** Thank you. As we have said in the previous comment, we have reviewed what was written in PROSPERO and we have made sure that all the information written in PROSPERO is reflected in the manuscript. In addition, we have added the missing information as we have pointed out in the previous comments. For example, we have added the following sentence:

“There were no restrictions regarding the date of publication of the study.”

Now, the paragraph about the "Search strategy" has this information:

“A protocol using the Meta-analysis of Observational Studies in Epidemiology standards (MOOSE) (34) was completed before beginning the literature search (S1). In order to offer an overview of the existing literature on SOC and its relationship with depression, we searched for: depression and sense of coherence (and MeSH terms). Then, the articles were classified by theme, and those related to chronic pain morbidity were selected. The search strategy was performed in this manner, since searching with the term chronic pain resulted in too many articles with keywords that were nosologic disorders related to chronic pain and were left out. Searches were carried out on PubMed, Web of Science, Embase, PsycINFO, Psicodoc, ScienceDirect and Dialnet. Details are registered at PROSPERO- ID = CRD42020157512. Retrieved articles from all databases were referenced in Mendeley Reference Management Software (Version 1.19.4, Mendeley Ltd.). Searches were conducted from November 01 to December 31, 2020. All papers were screened by title and full text for possible inclusion, after discarding duplicates. Studies from the accurate search should fully meet the following inclusion criteria: Individuals suffering from depression, adults (>18 years old) and both sexes. There were no restrictions regarding the number of participants or the date of publication of the study. Studies with underage individuals, non-indexed articles, systematic reviews and meta-analyses, theoretical studies, conference articles, insufficient data, single case studies and articles not written in English or Spanish were excluded. Fig 1 offers an extensive and accurate search flow chart.”

3) The format of Table 1 needs to be modified. The three--line table should be better.

**Answer reviewer 3:** Thank you. We have modified the format of the tables. We are open to any other modification that you consider appropriate.

4) The literature management software and data analysis software mentioned in the manuscript lack the necessary manufacturer information.

**Answer reviewer 3:** Thank you. We have added the following information:

“Mendeley Reference Management Software (Version 1.19.4, Mendeley Ltd.).” and “STATA SE software (version 16, StataCorp LLC)”.

Results

The format of Table 3 needs to be modified.

**Answer reviewer 3:** Thank you. We have modified the format of the tables. We are open to any other modification that you consider appropriate.

Discussions

The results in the manuscript are limited, but the authors seem to have discussed too much extraneous content. Some of the discussions lack the support of the results, more like some inferences. It is suggested to narrow the discussion and focus on the key factor of SOC.

**Answer reviewer 3:** Thank you. We have narrowed the discussion and we have eliminated much extraneous content, as suggested. Now the discussion is as follows:

"Our systematic review and meta-analysis pooled the data from 9 research articles. To the best of our knowledge, this is the first meta-analysis to study the association between SOC and depression in patients suffering from chronic pain. As for the main objective of our review, findings revealed that this association is yet to be explored. In the selection process, 149 articles were excluded, since they did not analyze the relationship between SOC and depression in chronic pain patients. Only 14 studies focused on chronic pain patients.

At first glance, the included studies indicate that SOC is an important factor in depression levels in chronic pain patients. The majority of the included studies reveal a negative association between SOC and depression symptoms (r = -0.55). Although the heterogeneity between studies was considerable, with the random‐effects meta‐regression models, we found that this heterogeneity was not due to differences in age and sex in the sample. Other studies have also found no differences regarding age, sex, ethnicity, nationality or study design (13). Therefore, the heterogeneity reported in this metanalysis could be due to the variety of illnesses that cause chronic pain (temporomandibular disorders (TMD), systemic lupus erythematosus (SLE), non-malignant chronic pain, different categories of chronic pain conditions, lumbar spinal stenosis (LSS), ankylosing spondylitis (AS), severe injuries following a life-threatening accident (SIAV), rheumatoid arthritis (RA) and non-metastatic breast cancer).

These results are quite in line with previous research conducted in the general population, in individuals with chronic comorbidity; burnout and caregivers’ burden; cancer; pregnancy and parenthood and neurodegenerative diseases. A quantitative study conducted in rural Crete (the SPILI III project; n = 195, mean age 67.2 ± 15.2) found that higher SOC scores are related to lower Beck Depression Inventory Scale (BDI) scores, indicating a lower frequency of depression in individuals with high SOC (19). Similarly, the results for a Swedish cohort study of adolescent females with and without major depressive disorders (MDD) and anxiety disorders (n = 139, aged 14 to 18-year-old) led the authors to suggest that the SOC scale may be an inverse measure of depressive symptoms (43). In a Finnish study (n = 4642, aged 25 to 74-year-old), a high inverse correlation was obtained between SOC and depression (r = -0.62). Also, SOC was positively associated and depressive symptoms and anxiety were negatively associated with various health-related measures (44). A study investigating SOC in community-dwelling older Spanish adults (n = 1106, aged 60 years and older) revealed that higher SOC was significantly associated with higher functional status and has a moderate correlation with depression (r = -0.47, p < 0.001) (45). In a sample of 548 Japanese students (age range 18–37 years), SOC was negatively associated with mental health (46). The results of a clinical trial with depressive patients suggest that both rehabilitation and conventional depression treatment in a first episode of depression may enhance the SOC and rehabilitation itself enhances SOC more effectively in those with less severe depression (47). SOC has also been found to be a key psychological resource supporting the resilience of caregivers and their ability to cope with stressors. This in turn protects their psychological health. SOC remained relatively stable over a one-year period (48). It also mediates factors related to postpartum depression (PPD), so that intervention for enhancing SOC is recommended for women at risk of PPD (49). Definitely, the stronger the SOC, the fewer the symptoms of perceived depression (10).

Focusing on the research analyzed, it is found that chronic pain patients having a low level of SOC have more depressive symptoms, higher pain rates and a poorer level of functioning, less acceptance and less adaptation ability (23–29). Only one study failed to uncover an association between SOC and depression (31). These results are in line with the literature on SOC and chronic pain. High SOC is related to lower mean scores in pain intensity (50) and a weak SOC increases the likelihood of sustained pain and functional problems (51).

SOC is an important health-promoting resource that induces a positive perceived state of wellbeing (13). Strong SOC also allows people to identify, use, and ‘recycle’ available resources and therefore, minimize feelings of tension and stress (52). So, a strong SOC improves one’s ability to deal with health problems (12,53,54). Some findings provide initial evidence that SOC is a significant unique predictor of symptom change (55). Individuals with a weak SOC appear to be less optimistic and display less learned resourcefulness, self-efficacy, hardiness, locus of control, mastery, self-esteem, acceptance of disability, and social skills (13). SOC has a mediating effect on the influence of positive psychological constructs, life experiences, and social systems on our well-being (56).

Adopting a systematic salutogenic orientation to focus on available cognitive and emotional resources, strengthen them and create new ones could be very useful (12,51). The results encourage further clarification of the role and use of SOC in the rehabilitation context to minimize feelings of tension and improve function in everyday life (52). Also, making individuals more resilient is of imperative need during the current pandemic situation and further research of the SOC issues in relation to depression seems to be a high priority."
